# PET/MRI-evaluated brown adipose tissue activity may be related to dietary MUFA and omega-6 fatty acids intake

**DOI:** 10.1038/s41598-022-08125-z

**Published:** 2022-03-08

**Authors:** Katarzyna Maliszewska, Edyta Adamska-Patruno, K. Miniewska, W. Bauer, M. Mojsak, A. Kretowski

**Affiliations:** 1 Department of Endocrinology, Diabetology and Internal Medicine, Medical University of Bialystok, M. Sklodowskiej-Curie24A, Bialystok, Poland; 2 Clinical Research Centre, Medical University of Bialystok, M. Sklodowskiej-Curie24A, Bialystok, Poland; 3 Independent Laboratory of Molecular Imaging, Medical University of Bialystok, ul. Żurawia 71A, 15-540 Bialystok, Poland

**Keywords:** Endocrinology, Gastroenterology

## Abstract

An investigation of new ways to activate brown adipose tissue (BAT) is highly valuable, as it is a possible tool for obesity prevention and treatment. The aim of our study was to evaluate the relationships between dietary intake and BAT activity. The study group comprised 28 healthy non-smoking males aged 21–42 years. All volunteers underwent a physical examination and 75-g OGTT and completed 3-day food intake diaries to evaluate macronutrients and fatty acid intake. Body composition measurements were assessed using DXA scanning. An FDG-18 PET/MR was performed to visualize BAT activity. Brown adipose tissue was detected in 18 subjects (67% normal-weight individuals and 33% overweight/obese). The presence of BAT corresponded with a lower visceral adipose tissue (VAT) content (p = 0.04, after adjustment for age, daily kcal intake, and DXA Lean mass). We noted significantly lower omega-6 fatty acids (p = 0.03) and MUFA (p = 0.02) intake in subjects with detected BAT activity after adjustment for age, daily average kcal intake, and DXA Lean mass, whereas omega-3 fatty acids intake was comparable between the two groups. BAT presence was positively associated with the concentration of serum IL-6 (p = 0.01) during cold exposure. Our results show that BAT activity may be related to daily omega-6 fatty acids intake.

## Introduction

Obesity and related metabolic complications are rising at a disturbingly fast rate worldwide^1^. Despite commonly known factors leading to weight gain, such as excessive food intake, a sedentary lifestyle, and the availability of pharmacological agents, the treatment of obesity remains challenging and is often unsuccessful. Obesity results in metabolic abnormalities, type 2 diabetes, and an increased risk of cardiometabolic complications^2^. Considering its negative effects on individuals, it is crucial to find new mechanisms to combat obesity.

Brown adipose tissue (BAT) has recently undergone investigation due to its unique ability to generate heat instead of storing energy^3^. In brown adipose tissue, lipids are packed in small droplets, unlike white adipose tissue (WAT), where triglycerides are stored in one large droplet inside the cell. BAT also has an abundance of large mitochondria and is more densely vascularized and innervated by the sympathetic nervous system compared to WAT^4^. Brown adipose tissue was initially thought to be present only in infants and children to maintain their core body temperature, but studies have also confirmed its presence in adults^5^—more commonly in lean individuals than in obese individuals^6^. The most known activators of brown adipose tissue are cold exposure and the agonist of the sympathetic nervous system (SNS)^7,8^. Brown adipose tissue can increase energy expenditure related to the function of unique uncoupling protein 1 (UCP1)^4^, which is considered to be a BAT indicator. A lower ambient temperature triggers the peripheral nervous system. Norepinephrine, released by the sympathetic nerve endings, induces intracellular triglyceride (TG) lipolysis during cold exposure. Brown adipocytes consume fatty acids and partially glucose, UCP1 is activated, and the process of non-shivering thermogenesis is initiated^9^.

In consideration of the uncomfortable and challenging aspects of cooling as well as the negative cardiovascular consequences of the pharmacological activation of SNS, the use of food ingredients is seemingly one of the more feasible methods of BAT activation^10^. Cold exerts its stimulatory effect on BAT through transient receptor potential channels, most of which are also receptors for various food products, such as capsaicin and its analogs, vanilloid^11,12^, which are representative agonists of the transient receptor potential vanilloid 1^13^.

The exceptional function of brown adipose tissue to increase energy expenditure is reflected in its protective role against obesity and type 2 diabetes mellitus^14,15^. Although the amount of BAT is reduced in obese individuals, as well as it is not present or not detectable in some other individuals, its activation in adults is viewed as a means to treat obesity. The thermogenic effects, effectiveness, as well as safety of some of these ways of BAT activation must be considered, and the risk and benefits balance need further investigation. The impact of dietary factors on BAT activity is still not clear^16^. However, some dietary ingredients, which seem to be safe and without any serious adverse effects, may impact BAT activation. The experimental studies with tea catechins have shown an increase in energy expenditure^17^ which suggests that tea catechins can activate and recruit BAT in humans. Also, direct stimulation of BAT activity (measured by imaging methods such as FDG-PET) was observed after the acute or chronic consumption of other bioactives in humans^18–20^. Interesting seems to be recently published data that provided clinical evidence for the impact of monounsaturated fatty acids (MUFA) on BAT activity. After 4 weeks of dietary intervention with olive oil, a significant increase of blood monounsaturated fatty acid levels was accompanied by increased BAT activity in lean but not in overweight/obese volunteers^21^. Some previously published paper also showed the favorable influence of polyunsaturated fatty acids (PUFA) omega-3 supplementation on BAT activity with beneficial effect on glucose homeostasis and insulin sensitivity^22–24^. A Mediterranean diet, which is in general rich in unsaturated fatty acids, has a beneficial effect on human health through several mechanisms, with the lipid-lowering effect as the most prominent^25^. The fatty acids are the main source of fuel used by activated brown adipocytes^26^. Therefore, their daily intake may be associated with BAT metabolism and activity, which was the hypothesis of our preliminary study. Another ambiguous question regards the role of the interleukin-6 (IL-6) in BAT activity. Studies in experimental animals indicate that IL-6 in the central nervous system partly mediates the suppression of food intake and may influence body weight^27^. Very interesting is also observation that concentration of IL-6 may be modulated by dietary consumption of mono- and polyunsaturated fatty acids^28–34^. The interleukin 6, mainly known as a proinflammatory cytokine and an anti-inflammatory myokine, seems to affect the activation of brown adipocytes^35^. Therefore, also this possible association between BAT and IL-6 concentration was tested in our study.

The aim of our study was to evaluate the activity of BAT in healthy males and the potential associations between BAT, resting energy expenditure, IL-6 concentration, and dietary intake.

## Results

**Figure 1:**
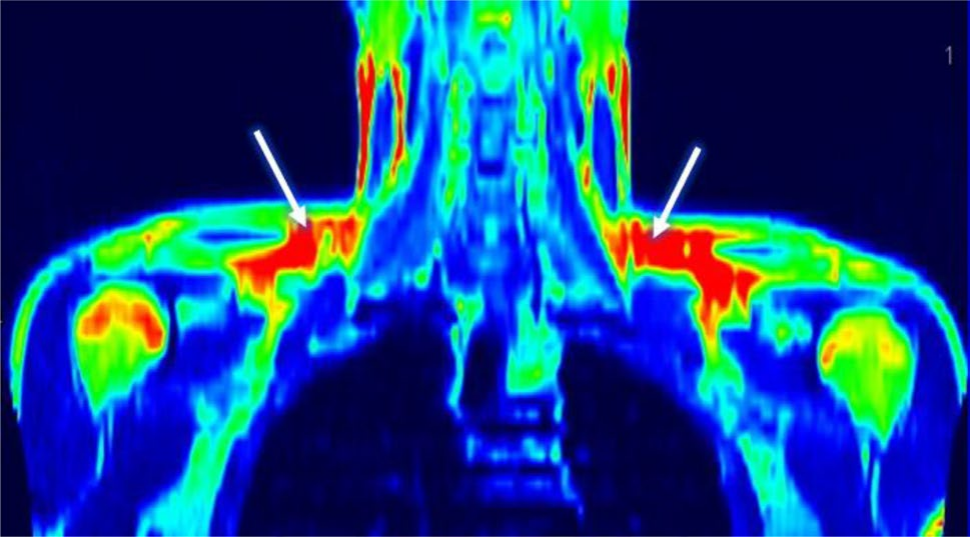
A typical PET scan of a BAT-positive subject. The arrows indicate brown fat depots in supraclavicular regions.

**Figure 2:**
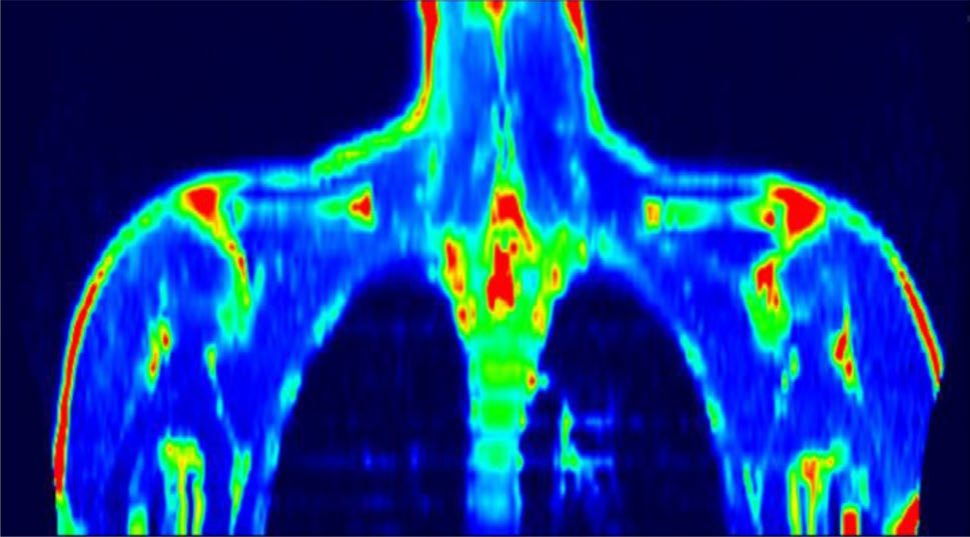
A typical PET scan of a BAT-negative subject.

In the studied group, brown adipose tissue was detected in 18 volunteers (BAT-positive) (Table 1), with a mean age of 24 years and a mean BMI of 25 kg/m^2^. A total of 67% of BAT-positive subjects had normal BMI, whereas 33% were overweight or obese. In 10 volunteers who underwent a 2 h cold exposure test, we did not observe the activity of brown adipose tissue in PET/MR images. These subjects were included in the BAT negative group (BAT-negative) (Figs. 1, 2).

**Table 1:** Characteristics of BAT-positive and BAT-negative group.

Variable	BAT-positive^1^	BAT-negative^2^	p-value^3^
N	18	10	
BMI < 25 kg/m^2^ (n, %)	12 (67%)	(40%)	
BMI > 25 kg/m^2^ (n, %)	6 (33%)	6 (60%)	
Age (years)	24.7 ± 2.4	30.3 ± 6.7	0.006
BMI (kg/m^2^)	25.65 ± 3.8	28.14 ± 4.2	Ns
VAT mass (g)	548.05 ± 515	1064.7 ± 782	0.02
VAT AT (%)	2.2 ± 1.19	3.8 ± 1.9	0.007
VAT BW (%)	0.5 ± 0.4	1.0 ± 0.67	0.01
VAT volume (cm^3^)	609 ± 545	1595 ± 838	0.02
A/G ratio^4^	1.0 ± 0.15	1.2 ± 0.24	0.02
MUFA intake (g/day)	27.9 ± 8.2	37.35 ± 9.2	0.02
PUFA intake (g/day)	10.6 ± 2.8	12.5 ± 9.2	Ns
Omega-6 intake (g/day)	7.2 ± 2.37	10.2 ± 2.9	0.03
Omega-3 intake (g/day)	2.0 ± 0.99	2.9 ± 1.0	Ns
IL-6 V2^5^ 0 min (pg/ml)	756.8 ± 272	650.0 ± 215	Ns
IL-6 V2^5^ 60 min (pg/ml)	926.35 ± 205	818.79 ± 316	0.01
IL-6 V2^5^ 120 min (pg/ml)	970.4 ± 170	865.78 ± 308	0.01
IL-6 V2^5^ 240 min (pg/ml)	911.88 ± 202	943.63 ± 375	Ns
IL-6 AUC V2^5^ (pg/ml × 240 min)	72,709 ± 22,186	56,934 ± 21,496	0.07
AUC REE adjusted^6^ (kcal/min/ffm × 120 min)	2.3 ± 0.4	2.2 ± 0.6	Ns
Δ REE^7^	258.7 ± 289	204 ± 384	Ns
Energy intake kcal	2149 ± 505	2171 ± 321	Ns
Carbohydrate intake (g)	270 ± 70	253 ± 46	Ns
Protein intake (g)	119 ± 41	98 ± 27	Ns
Fat intake (g)	72 ± 21	87 ± 21	Ns

The parameters in the table are represented by its means and standard deviations. ^1^BAT-positive—a group of subjects in whom brown adipose tissue was detected; ^2^BAT-negative—a group of subjects without detectable brown adipose tissue; ^3^p value—adjusted for age, daily average kcal intake, DXA Lean mass; ^4^A/G ratio, android to gynoid ratio; ^5^V2—the second visit—cooling exposure with PET-MR; ^6^AUC whole-body energy expenditure adjusted for age, daily kcal intake, DXA Lean mass, ^7^a change of whole-body resting energy expenditure between 0 and 120 min of cold exposure; *BMI* body mass index, *VAT mass* the visceral adipose tissue mass, *VAT AT (%)* the visceral adipose tissue percentage of adipose tissue, *VAT BT (%)* the visceral adipose tissue percentage of body weight, *VAT volume* the visceral adipose tissue volume, *MUFA* Monounsaturated Fatty Acid, *PUFA* Polyunsaturated Fatty Acid, *IL-6* Interleukin 6.

In the BAT-positive group, the mean volume of brown adipose tissue was estimated as 26 355 ± 43 202 mm^3^ and the mean activity of BAT was 19.1 ± 4.3 µmol × (100 g^−1^) × min^−1^. We noticed a lower mass and volume of visceral adipose tissue (p = 0.02, after adjustment for age, daily kcal intake, and DXA lean mass) in the BAT-positive group, as well as a lower percentage of VAT content (p = 0.007, after adjustment for age, daily average kcal intake, and DXA lean mass). No significant association was found linking BAT volume and 18F-FDG uptake with fat-free mass (FFM) (p = 0.57) and lean mass (lean mass) (p = 0.54). We did not observe any differences between studied groups in FFM, lean mass, fat mass, and OGTT results (data not shown).

**Figure 3:**
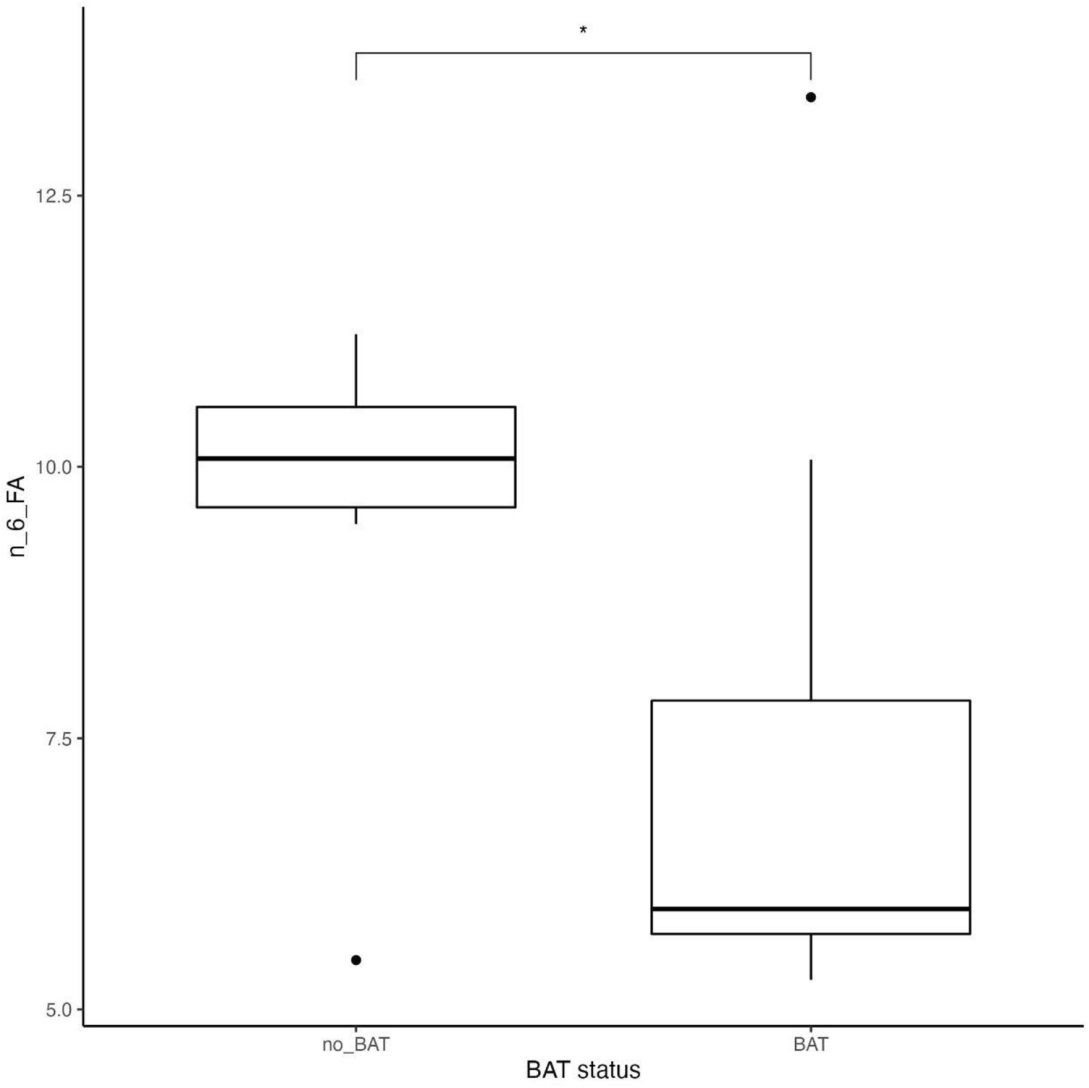
Omega-6 FA intake in subjects dependently on BAT status (p = 0.03)*.

**Figure 4:**
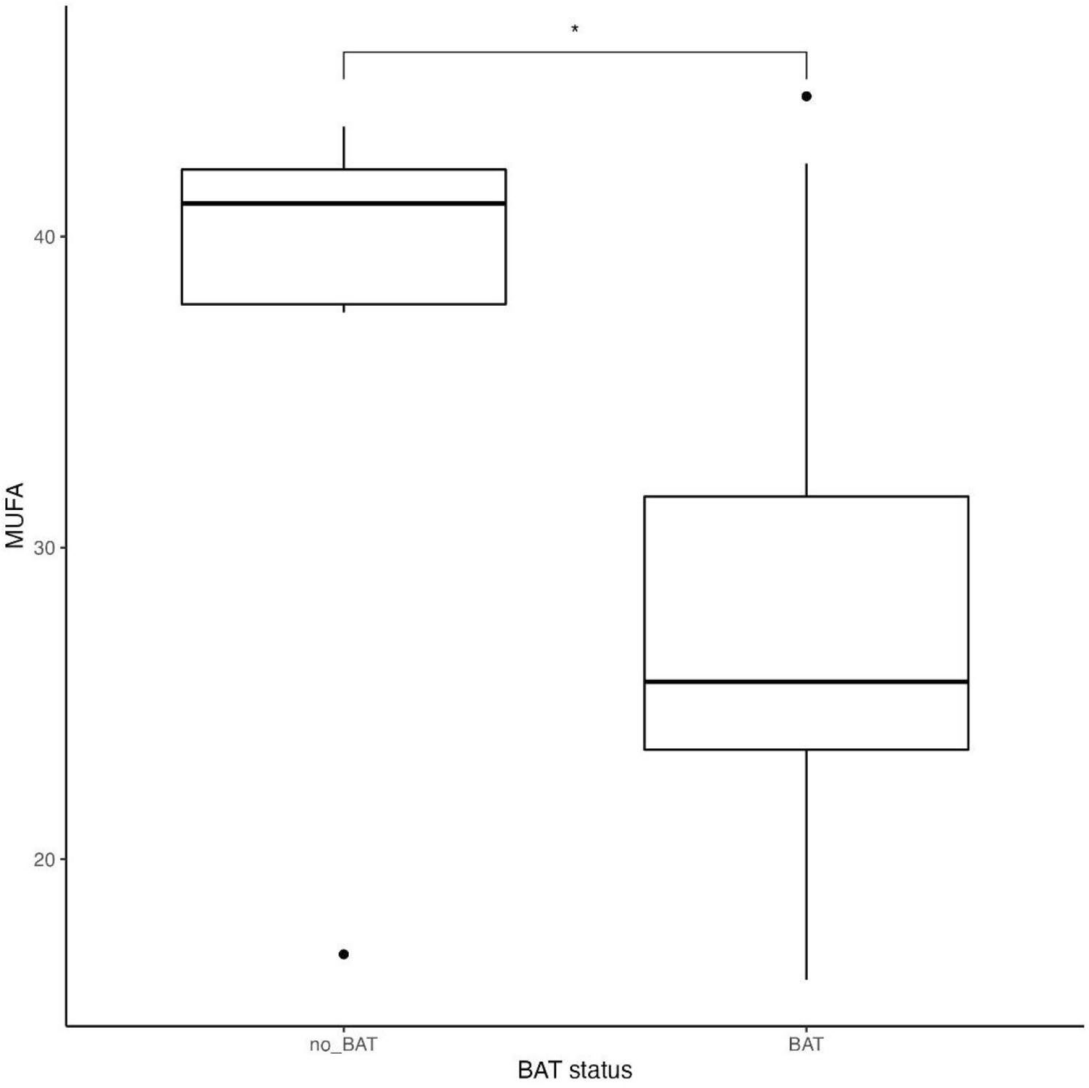
MUFA intake in subjects dependently on BAT status (p = 0.03)*.

**Figure 5:**
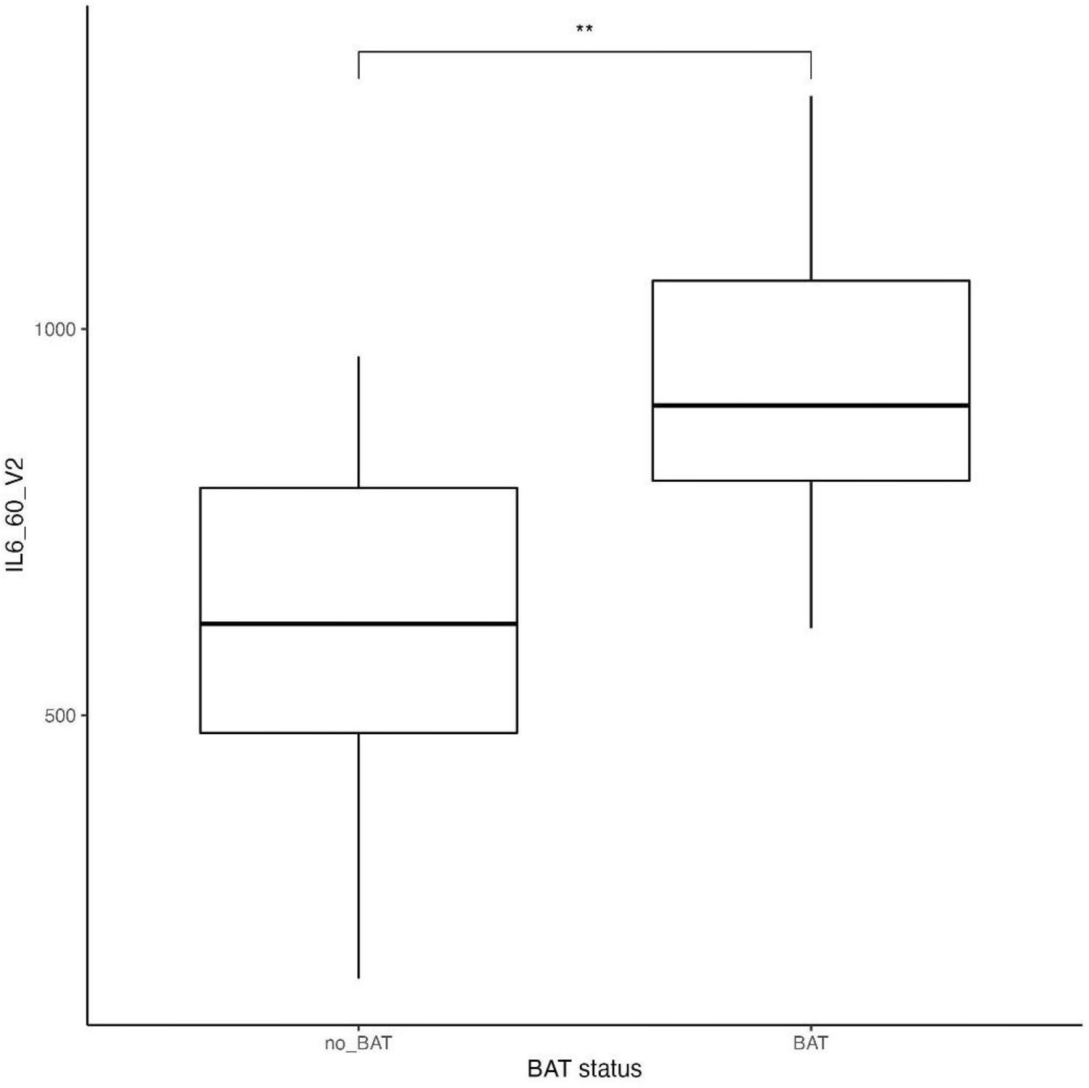
IL-6 concentration in serum during cold exposure in subjects dependently on BAT status (p = 0.01)**.

We also observed that omega-6 fatty acids intake and MUFA intake was significantly lower in BAT-positive subjects (p = 0.03 and p = 0.02, respectively, Table 1) (Figs. 3, 4), compared to BAT-negative, after adjustment for age, daily average kcal intake, and DXA Lean Mass, whereas omega-3 fatty acids intake did not differ significantly between the studied groups. We did not discover any differences between groups BAT-positive and BAT-negative in the total kcal energy intake and consumption of carbohydrates, protein, and fat (data not presented). Also, BAT-positive group was characterized by a higher concentration of IL-6 during 2 h cold exposure (p = 0.01 after adjustment for age, daily average kcal intake, and DXA Lean mass) (Fig. 5).

Multiple linear regression models were applied to test independent determinants of the BAT-positive group. Generally, the presence of brown adipose tissue was negatively associated with omega-6 fatty acids intake (Est. − 2.96, R^2^ = 0.54, p = 0.003, after adjustment for age, daily kcal intake, and DXA Lean Mass), PUFA intake (Est. − 2.13, R^2^ = 0.39, p = 0.05), and MUFA intake (Est. − 7.42, R^2^ = 0.55, p = 0.03, after adjustment for age, daily average kcal intake, and DXA Lean mass). It was positively associated with the concentration of serum IL-6 (Est. 330,64, R^2^ = 0.32, p = 0.05, after adjustment for age, daily kcal intake, and DXA Lean mass) during cold exposure.

The BAT volume was positively associated with omega-3 fatty acids intake (Est. 0.00001, R^2^ = 0.69, p = 0.00007) and long-chain PUFA intake (Est. 0.0001, R^2^ = 0.38, p = 0.0005). It was negatively associated with an omega-6/omega-3 fatty acids ratio (Est. − 0.00002, R^2^ = 0.53, p = 0.001, after adjustment for age, daily kcal intake, and DXA Lean mass). We did not notice any differences in the AUC of REE measurements during cold exposure tests, except for the slight tendency (p = 0.07) of a higher Δ of REE between 0 and 120 min of the cold exposure test in BAT-positive subjects.

## Discussion

In our study, we evaluated the relationship between dietary intake and the activity of brown adipose tissue in healthy males aged 21–42. Subjects in which the presence of BAT was found presented a lower daily consumption of MUFA and PUFA omega-6 fatty acids. Moreover, the BAT volume was related to higher omega-3 fatty acids intake, long-chain PUFA, and a lower ratio of omega-6/omega-3 fatty acids. A lower percentage of visceral adipose tissue was observed in subjects with detectable brown adipose tissue, along with a higher serum IL-6 concentration during the cold exposure test. This finding is consistent with previously published studies^36^. However, Monfort-Pires et al.^21^ reported that MUFA-rich olive intake induces the activation of BAT, which is in contrary to our observation; nevertheless, it has been noted only in lean subjects.

Our analysis of dietary omega-3 and omega-6 fatty acids intake produced valuable insight as well. Notably, omega-3 and omega-6 fatty acids belong to polyunsaturated fatty acids. The proportion between omega-3 and omega-6 fatty acids is crucial for health, and the proper ratio should be 1–4:1 of omega-6 to omega-3 fatty acids^37^. In all diets, especially Western diets, omega-6 fatty acids cover the majority of PUFAs food supply. Moreover, dietary changes over recent decades have resulted in a significant increase in the intake of omega-6 fatty acids, thus altering the omega-6 to omega-3 fatty acids ratio to around ~ 15:1^38^. An imbalance in the omega-6/omega-3 fatty acids ratio may enhance an immune response. Omega-3 fatty acids are considered to be an anti-inflammatory agents, while omega-6 fatty acids have an opposite function. Derivatives from both polyunsaturated fatty acids—eicosapentaenoic acid (EPA), which belongs to omega-3 fatty acids, and arachidonic acid (ARA), which belongs to omega-6 fatty acids—compete for the same enzymes in the prostaglandin biosynthesis^39^. The long-chain omega-3 fatty acids depressed the production of proinflammatory prostaglandin^40^, while the omega-6 fatty acids are known for their proinflammatory properties. ARA can be metabolized to prostaglandins (A2, E2, I2, and thromboxane A2) by cyclooxygenases-2 (COX-2), while leukotrienes (B4, C4, and E4) are biosynthesized from ARA by lipoxygenases (5-LOX)^41^. The influence of omega-3 and omega-6 fatty acids on the immune system has been widely surveyed^42^. PUFA show also a significant impact on the regulation of adipocyte differentiation and their function. The beneficial role of the omega-6 and omega-3 fatty acids and long chain PUFA dietary intake on adipose tissue development and function has been already shown^43^ Diet enriched in particular in the omega-3 PUFA, may decrease adipose tissue content, however, the physiological and cellular effects of PUFA may depend on many factors, and it has been noted that omega-6 PUFAs may exert either an anti- or a proadipogenic effects^44^. Recently, studies on animal models reported that high-fat diets rich in PUFA affect the expression of uncoupling protein 1 mRNA in brown adipose tissue. The increase was more significant with the supplementation of omega-3 PUFA than with omega-6 PUFA^45^. In line with previous results, outcomes from the intervention study with supplementation with omega-3 long-chain PUFA showed that omega-3 fatty acids enhanced thermogenesis via the activation of brown adipose tissue (BAT)^46,47^. A recently published paper on metabolite profiling by liquid chromatography-mass spectrometry (LC–MS) in humans with detectable BAT showed a unique systemic PUFA and oxylipin profile with increased levels of anti-inflammatory omega-3 fatty acids^48^. The above-listed papers supported the relationship between PUFAs and brown adipose tissue. We did not notice any differences in omega-3 fatty acids intake between subjects with and without identified BAT activity, but the linear regression models showed that the BAT volume was positively associated with omega-3 fatty acids intake. Moreover, as mentioned above, the proper ratio between omega-6 and omega-3 fatty acids may also play a crucial role in metabolism^49^. A lower dietary omega-6/omega-3 fatty acids was noted to improve the thermogenic response of BAT and WAT under β3-adrenergic stimulation^50^. Based on our results, we can hypothesize that a lower amount of omega-6 fatty acids in a person’s diet may have a beneficial effect on brown adipose tissue activity, possibly due to the enzyme competition between omega-3 and omega-6 fatty acids, which need further investigation. Dietary omega-6 fatty acids intake could be one of the potential mechanisms underlying the activity of brown adipose tissue.

PUFA and their metabolites may have an impact not only on the BAT activity but on the conversion of white into brite adipocytes as well^51^. It was noted that diets rich in ARA favor WAT formation by preventing the “browning” process^52^. However, recently published results suggest no effect of dietary fish oil supplementation on the recruitment of brown and brite adipocytes in mice or humans under thermoneutral conditions^53^. The thermoneutral conditions could be an explanation of noted conflicting results, since different circulating PUFA and oxylipins (being the lipid mediators produced from PUFA) profiles in BAT-positive and BAT-negative subjects were noted^54^ and cold exposure significantly increased plasma lipid composition only in BAT-positive individuals, strongly supporting the relationship between BAT and PUFA. The presence of BAT was also characterized by increased concentrations of omega-3 fatty acids and their precursor molecules^54^.

We did not notice any differences between the studied groups in regard to total energy and macronutrient intake. Our results are in line with Sanchez-Delgato et al.’s study in which associations between BAT volume or 18F-FDG uptake and energy intake, assessed via either the ad libitum meal or the habitual dietary intake, were not observed^55^.

In our study, we also observed that subjects with detectable brown adipose tissue are characterized by lower visceral adipose tissue and lower BMI. The fact that individuals with identified BAT activity were significantly younger could also affect this observation. Nevertheless, our results are in line with Matsushita’s study, which similarly reported that subjects with BAT are younger and have less abdominal fat^56^. Several studies observed a relationship between body composition, adiposity-related parameters, such as BMI, central body fat distribution, and BAT, thus indicating a reduced amount of brown adipose tissue in obese subjects^57–59^. Obesity is characterized by a chronic low-grade inflammatory state in adipose tissue maintained by the secretion of a wide range of inflammatory proteins. Systemic inflammation, especially TNF alfa, suppresses the thermogenic activity of brown fat’s capacity to reduce energy expenditure^60^. Moreover, data suggested its contribution to the whitening of BAT that occurs after the prolonged consumption of high-fat foods^61^. In the 52-week-old insulin receptor knockout mice, a significant decrease of BAT mass was observed with a significant increase of visceral WAT mass compared to 33-week-old mice^62^.

We observed a slight tendency of higher Δ of the resting energy expenditure (REE) between baseline and 120 min of the cold exposure in BAT-positive subjects, but we did not observe any differences between BAT-positive and BAT-negative individuals in the REE during the cold exposure test. These results correspond with the outcomes of Orava et al.’s research^59^.

In our study, we noticed an increase of serum IL-6 during 2 h of cold exposure, which is in line with the results of other authors^63^. It may seem confusing, as IL-6 is known as a proinflammatory cytokine, while its effect and associations with BAT need more investigations since it is suggested that IL-6 may increase its activity^64^. The lack of IL-6 expression impaired the beneficial effects of BAT transplantation on metabolic health through the interaction with FGF21^65^. Additionally, IL-6 is indispensable for the induction of WAT browning in response to a cold environment^66^. Moreover, another anti-inflammatory role of interleukin-6 has also been shown. It is released by skeletal muscle in response to exercise and promotes insulin sensitivity^67^.

To the best of our knowledge, our study is one of the first to assess the relationship between daily nutrient intake assessed by the 3-day food diary and the activity of brown adipose tissue. The relationship between diet-induced thermogenesis has been evaluated in the interventional studies^68–70^. It is worth highlighting the significant outcomes from a systematic review (PROSPERO) and meta analyzes which showed no differences in standardized uptake value of BAT following a single meal or after 6 weeks of l-Arginine supplementation. Resting energy expenditure, however, was increased following a single meal and after supplementation of capsinoid and catechin when compared to a control condition^16^. The topic is still relevant and needs to be further investigated. The results from our study indicate an association between BAT volume/activity and omega-3 and omega-6 polyunsaturated fatty acids. Moreover, the results suggest that further attention should be directed toward the right balance between omega-6 and omega-3 fatty acids in brown adipose tissue activity. Researchers should evaluate whether polyunsaturated fatty acids directly influence the activation of BAT or if they indirectly do so through the beneficial effect of omega-3 fatty acids on body fat, weight loss, or the reduction of an inflammatory state^71–73^. Maintaining an adequate proportion of body fat with a normal body index may promote the activation of brown adipose tissue. Future studies should investigate how do omega-3 and omega-6 fatty acids activate BAT, if directly or through particular mechanisms.

It is worth to notice also, that previous research on BAT primarily used PET/CT as a tool for imaging human brown adipose tissue^74^. PET/MR is the preferred imaging source because of the lack of ionizing radiation, feasibility, and higher spatial resolution. PET/MR imaging has previously been used to detect the presence of BAT in adults as well as in children^75,76^.

Our study has some limitations. The major limitation is a relatively small sample size, but the number of subjects enrolled in the study is comparable to the previous survey^77^. The main reason of limitation for conducting a large-scale trial is the high costs associated with PET/MR scanning and a tracer purchase. Therefore, if possible, our findings should be further tested in a larger population and different ethnic groups. The other important fact is that in our study, only the BAT glucose uptake was measured, and it is important to note that the main source of energy for BAT are fatty acids^78,79^. Therefore, we overlook the possibility that some of the BAT-negative subjects, defined by the glucose rate, might have a significant fatty acid uptake by the BAT tissue. Moreover, FDG allow to analyze and to localize BAT but it could be less informative for BAT activity. Indeed, BAT thermogenic activity, as mentioned above, is mainly due to fatty acid oxidation and uptake^77^. Because of the difficulties associated with obtaining a tracer to investigate fatty acids metabolism in humans, we used 18F-FDG in our study. Moreover, brown adipocytes are interspersed within white adipose tissue. Therefore, through PET detection, BAT regions could contain both, BAT and some white adipocytes^57^. It is also possible that the cooling was not optimal for some of the subjects, especially for those who were obese, thus resulting in false-negative results related to BAT activity. In our study, the water-perfused blankets were used, and the many cold exposures to large skin areas, such as via water-perfused suits or vests, seem to demonstrate minor variation in BAT activation^80,81^, what also should be considered.

## Conclusions

In conclusion, we noted lower visceral fat accumulation in subjects with identified BAT, which confirms the protective role of brown adipose tissue and indicates that BAT shows strong potential as a means to combat obesity and its metabolic consequences. Moreover, our results suggest that both, dietary MUFA, as well as omega-3 and omega-6 fatty acods intake, may be associated with the volume and activity of BAT in healthy males aged 21–42, which deserve further investigations.

## Materials and methods

### Study participants

The study group comprised of 28 healthy, non-smoking Caucasian males aged 21–42 years (mean age 26.75 ± 5.11 years old). Sixteen participants had normal body weight (BMI < 25 kg/m^2^) and 12 were obese/overweight (BMI > 25 kg/m^2^). Volunteers to this study were recruited from the other cohort study group, described in detail previously^82,83^. The participants in this study were without any comorbidities (e.g., hypo- or hyperthyroidism, asthma, cardiovascular disease, renal or liver failure, and any acute or chronic diseases) and were not taking any medications (e.g., beta-blockers) or dietary supplements that could have had an impact on the results. Outside and shift workers were excluded from the study as well. Subjects were enrolled in the study, and all study procedures were performed during the October–April periods of 2016–2018.

### Screening of subjects

During the screening visit, the medical history and metabolic status of all volunteers were reviewed. They underwent a physical examination, routine laboratory tests (hematology, TSH, creatinine, liver enzymes, Na, K, CRP), blood pressure measurement, an electrocardiogram (ECG), and an oral glucose tolerance test (OGTT). The OGTTs were performed according to World Health Organization (WHO) recommendations, with a 75-g glucose load.

### Dietary assessments

All subjects completed a 3-day food diary. Subjects were asked to compare their portion sizes with each portion size’s color photographs from “Album of Photographs of Food Products and Dishes” developed by the National Food and Nutrition Institute^84^ and weigh food, if possible. Subjects were asked to record the amount and the type of fats and oils used for cooking as well. Daily total energy, macronutrients, monounsaturated fatty acids (MUFA), polyunsaturated fatty acids (PUFA), and omega-3 and omega-6 fatty acids intake were analyzed using Dieta 6 software (National Food and Nutrition Institute, Warsaw, Poland), which was developed and which is continuously updated by the National Food and Nutrition Institute (Warsaw, Poland). This software is used to calculate the nutritional value of food and diets based on tables of the nutritional value of local food products and dishes and is commonly used to evaluate the fatty acid dietary intake^85^.

### Anthropometric measurements

The body height and weight of participants were measured using a standardized method. Bodyweight was measured in a standard way (InBody 220, Biospace, Korea). Body composition and body fat distribution measurements were assessed using DXA scanning (enCORE™, iDXA Lunar GE Healthcare). In further analyses, the following parameters were evaluated: visceral adipose tissue mass (VAT mass), visceral adipose tissue volume (VAT volume), the visceral adipose tissue percentage of body weight (VAT BW %), the visceral adipose tissue percentage of adipose tissue (VAT AT%), the android fat to gynoid fat ratio (DXA A/G ratio), free fat mass (FFM), and lean mass (Lean mass).

### Cold exposure and PET/MR scanning

During the second visit, in the fasting state, all volunteers underwent 2 h of cold exposure. Water perfused blankets were used as part of the applied protocol for cooling. Blood samples were also taken in the 60th and 120th min of cooling. After this procedure, a fluorodeoxyglucose F 18 injection (18F-FDG) (4 MBq/kg of body mass) was given, and a PET/MRI scan (Biograph mMR 3 T, Siemens Healthcare, Erlangen, Germany) of the whole body was performed during the autumn and winter periods.

Regions of interest (ROIs) were manually outlined in fusion images composed of a summed dynamic 18F-FDG PET image and magnetic resonance (MR). The software Carimas, developed at the Turku PET Centre in Finland, was applied for the image analyses. ROIs were drawn in image planes with a defined structure of brown adipose tissue and in the aortic arch in the time frame with the highest first-pass concentration of the tracer. Regional time-activity curves (TACs) were generated, and glucose uptake rate data for the regions were assessed. The influx rate constant (Ki) of FDG-F18 for BAT was determined using the Gjedde-Patlak model. A lumped constant (LC) value of 1.14^86^ was used for all adipose tissues. The glucose uptake rate was calculated as follows: plasma glucose concentration × Ki × LC^−1^. The activation of BAT was defined as a glucose uptake rate higher than 2.0 µmol × (100 g^−1^) × min^−1^, which was chosen after a visual interpretation of PET images and the determination of the BAT glucose uptake rate at warm conditions, where it was always lower than 1.7 µmol × (100 g^−1^) × min^−1^^87^. Individuals in which BAT was detected were matched to the BAT positive group (BAT-positive), while subjects without detectable BAT in PET/MR images were classified as BAT negative (BAT-negative).

### Resting metabolic rate measurements

During the cold exposure, whole-body resting energy expenditure (REE) was assessed using a computed open-circuit indirect calorimetry method based on the consumption of O_2_ and the production of CO_2_. The 30-min long measurements of resting oxygen uptake and resting carbon dioxide production were performed using a ventilated canopy Vmax Encore 29n system (Viasys HealthCare, Yorba Linda, CA, USA) at the baseline (− 30 to 0 min) and every 30 min until 120 min of cold exposure.

### Blood collection and biochemical measurements

During the cold exposure, blood samples were collected and stored at − 60 °C for further analyses. The serum IL-6 concentration was determined using an enzyme-linked immunosorbent assay (ELISA) (ELISA Kit for Human Interleukin 6 (Human IL-6); R&D Systems, Inc., Minneapolis, MN 55413, Canada, D6050) according to the manufacturer’s protocol and based on observing the principles of internal laboratory control for the performed determinations. The serum glucose level was measured using the colorimetric methods of the Cobas c111 analyzer (Roche Diagnostics, Basel, Switzerland). Samples and controls were measured in the same run using the blind analysis method.

### Statistical analyses

Numerical data were summarized with a number of observations (N), arithmetic means, and standard deviations (SD). For categorical data, the number of observations and frequencies were presented. Study participants were divided into two groups based on the presence of brown adipose tissue: BAT-positive and BAT-negative. Continuous parameters were examined for normality with the Shapiro–Wilk test and thorough visual inspection. The homogeneity of variance across groups was studied using the Levene test. Non-parametric tests were used for response variables that failed these two tests. The differences between the selected responses and BAT groups were then compared using an analysis of variance (ANOVA) or the Kruskal–Wallis test for numerical variables, with, respectively, a Tukey or Dunn post hoc test with a Holm p-value adjustment (in case multiple pairwise tests were performed or when there were multiple grouping variables). In order to study the hypothesis that there is a significant association between the presence of brown adipose tissue and body composition, as well as the hypothesis that the average daily consumption of omega-3 and omega-6 fatty acids can significantly alter brown adipose tissue activation, we studied its association using multivariate linear regression models. In all two-group comparisons and regression models, an adjustment for age, daily average energy intake (kcal/day), and DXA lean mass) was made to eliminate the potential effect of the covariates. The statistical significance level was set at 0.05 for all two-sided tests. All calculations were prepared in R (R version 4.0.2)^88^.

### Ethics

The local Ethics Committee of Medical University of Bialystok, Poland (R-I-002/233/2015) approved the study protocol, and written informed consent was obtained from all participants. The procedures were performed in accordance with the Helsinki Declaration of 1975 as revised in 1983.
